# Concerns with oral health care services for adults with cognitive and intellectual disabilities

**DOI:** 10.6026/97320630016974

**Published:** 2020-12-31

**Authors:** Meignana Arumugham Indiran, Aravind Kumar Subramanian, Jayashri Prabakar, R Pradeep Kumar, D Sri Sakthi, L Leelavathi

**Affiliations:** 1 Department of Public Health Dentistry, Saveetha Dental College, Saveetha Institute of Medical and Technical Sciences, Saveetha University, Chennai 77, India; 2 Department of Orthodontics, Saveetha Dental College, Saveetha Institute of Medical and Technical Sciences,Saveetha University, Chennai 77, India

**Keywords:** Oral health, dental utilization, barriers, Adults, intellectual disabilities, cognitive disabilities

## Abstract

It is of interest to document data on oral health care services for adults with cognitive and intellectual disabilities. Hence, a study protocol was registered at the International Prospective Register of Systematic Reviews (PROSPERO) with registration number: CRD42020150759. We used PubMed, Science Direct, LILACS and SCIELO to collect data from known literature using keywords containing MESH (Medical Subject Headings) terms. The risk of bias rating for the collected data was calculated using the Newcastle-Ottawa assessment Scale. The AHRQ (Agency for Healthcare, Research and Quality) was used for classifying the level of evidence in the collected data. Analysis of available data shows that there is a lack of dentists with adequate skills to treat people with disabilities resulting in high cost for dental treatment. Thus, we conclude that inconvenient location of dental clinic, lack of dentists willing to treat people with disabilities and attitude of dental staff towards people with learning disabilities were considered as barriers and challenges faced for dental health service utilization in this context.

## Background

Oral health has an important influence on the psychological and social health of individuals [1]. The consequences of poor oral health are toothache, difficulty in eating, decreased self-esteem, impaired social interactions. This affects the quality of life and general health in aged people [2-8]. Disability is the complex interactions between health conditions, environmental and personal factors according to the Disability and Health records in the International Classification of Functioning (ICF) by World Health Organization (WHO) [9]. Intellectual disabilities (ID) refer to personal factors, health condition, and environment effects on individuals [9]. A significant intelligence impairment; a reduced adaptive behaviour/social functioning; is common among ID people and the condition develops even before the age of 18 [10]. Cognition refers to mental processes, including attention, memory, producing and understanding language, solving problems, and decision-making [11]. Individuals with ID are particularly more prone to poor oral health and have more complex oral health care needs [12,13]. This inequality is due to the barriers to accessing quality health care, behavioural and communication challenges and a higher likelihood of having lower educational and income levels [14-16]. Anders and Davi reported that people with ID have a higher prevalence of untreated dental caries and periodontal disease [17]. Poor general health includes aspiration pneumonia and major chronic diseases such as cardiovascular disease, diabetes, respiratory disease and stroke [18-24]. Numerous factors such as socio-demographic characteristics, poor oral health, cognitive impairment and intellectual disability are considered to be associated with dental care utilization among adults [25-27]. Gilbert GH et al., reported that individuals with a greater deficit in cognitive functioning were less likely to have dental care [28]. Walsh et al. showed that individuals with relatively low or moderate cognitive function were significantly less likely to have visited the dentist compared to those with higher cognitive function [29]. Therefore, it is of interest to document data on Oral health care services for adults with Cognitive and Intellectual disabilities.

## Methodology

### Design:

Systematic review of population-based survey data was used in this study.

### Protocol:

The study protocol was registered at the International Prospective Register of Systematic Reviews (PROSPERO) with Registration number:CRD42020150759. This study includes five stages: (1) Identifying the research question; (2) Literature search; (3) Study selection; (4) Data extraction; and (5) Summarizing and reporting the results.

### Parameters for PICO Analysis

Population: Adults

Problem: Cognitive disabilities

Outcome: Barriers to Oral health care services with utilizations

### Eligibility Criteria:

The factors associated with utilization and barriers to oral health care services by adult participants were considered. Data on study participants with cognitive impairment and learning disabilities from known literature were used.

### Search methodology:

We used PubMed, Science Direct, LILACS and SCIELO to collect data in this regard for keywords containing MESH (Medical Subject Headings) terms (Table 1 see PDF).

### Data selection:

Difference of opinion was resolved by discussing with the third reviewer (ARV). Quality Assessment criteria to evaluate data were carried out using Newcastle-Ottawa Scale guidelines [30]. The Newcastle-Ottawa Scale utilizes a star assessment system, where in stars were allocated across three domains, including five stars for participant selection, two stars for comparability and three stars for measurement of outcome. If fewer than six stars were scored, the study was considered to be at a high risk of bias. The risk of bias was independently assessed manually and conflicts concerning risk of bias were sorted by discussion. The AHRQ (Agency for Healthcare and Research and Quality) [31] was used for classifying the level of evidence. This system employs seven levels: 1) systematic review or meta-analysis; 2)randomized controlled trials; 3) controlled trials without randomization; 4) case-control and cohort studies; 5) systematic reviews of descriptive and qualitative studies; 6) single descriptive or qualitative study; and VII) opinion of authorities and/or report of expert committees.

### Data extraction:

Data were extracted independently as shown in Table 2 (see PDF).

### Note on statistical analysis:

Pooling of data was not possible due to hetero-genous nature of the selected information.

## Results and Discussion:

We used PubMed, Science Direct, LILACS and SCIELO to collect data in this regard for keywords containing MESH (Table 1 - see PDF). Thus,259 articles were selected from databases after removing redundant data. Data was further gleaned using criteria as shown in Figure 1(see PDF). Description of literature data and levels of evidence according to AHRQ is given in Table 2(see PDF). Data on the risk of bias (quality assessment of studies using new castle-ottawa scale) is given in Table 3(see PDF) and Figure 2(see PDF). Data shows that under the domain selection, one study [33] received the maximum 5 stars, whereas 2 studies [32,36] received 4 stars and 3 studies [34, [35, [37] received 3 stars. All 6 studies received a maximum of 1 star [32-37] for the domain comparability. Based on outcome assessment, all the 6 studies received a maximum of 1 star [32-37]. All the six reports exhibited a total score of 6 or more, which indicates that there was no high risk of data bias.

The importance of barriers to oral health care services varies exponentially with respect to various population segments. Barrier to dental care occurs for functionally dependent individual and the functionally independent person and people with disabilities residing at home or institutions. According to Penchansky and Thomas [38] the concept of access to dental care includes availability, accessibility, accommodation, acceptability, and affordability. When combined with Maxwell's [39] dimensions of health care quality (effectiveness, efficiency, equity, access, acceptability, and appropriateness) a more practical and applicable version of access is explained that can be implemented to any health care, particularly for those with learning disabilities. The findings of the present systematic review illustrated that oral health care services for people with cognitive and learning disabilities varied exponentially. In study conducted by Bojan B. Petrovic et al. [33] described a situation of poor oral health status in a group of persons with ID, with a significant proportion of persons with untreated decay, poor oral hygiene and manifest gingival inflammation and almost 50%-80% ID persons experienced difficulties with oral health care delivery [40]. Oral care utilization rate decreased with increased age and disability severity [33] and Cognitive function was the most highly correlated with dental care utilization in the study sample [32]. There are few concerns, which require more attention with respect to providing oral health care services to individuals with learning disabilities. They are attitudes of dental staff or dentists towards people with learning disabilities; the lack of collaboration between and within services; the lack of continuity of care and choice; and a failure to acknowledge that people with learning disabilities have the same rights to treatment as others. As a result, the care received by individuals with learning disabilities did not meet the aspirations of valuing people and valuing people's oral health [41].

Dental cost, location of dental clinic, lack of dentists prepared to treat people with disabilities, transportation problems and lack of comprehensive oral health care were reported as a barrier in accessing dental care [34, 36]. Information on barriers is very important for planning effective strategies to enable access to oral health services. Training of dentists at under- graduate and postgraduate level and Dental hygienists in providing oral health care to people with special needs at a professional level should include interdisciplinary and inter professional team experiences, with an emphasis on oral health promotion and disease prevention, so they are competent and willing to provide preventive services and, if necessary, treat people with disabilities [34]. Significantly fewer young adults with Intellectual Disability (45%) visited a dentist at least once per year, compared with those without Intellectual Disability (58%). Intellectual Disability severity and the presence of co-occurring developmental disabilities predicted dental care use. Socio-demographics, daily functioning, societal participation, dental services, and dental health factors were examined as predictors of dental care frequency [37]. Applying a bottom-up approach to dental services means that planning would start with the person, finding out their needs and preferences, and then fitting service delivery around the person, rather than utilising the present method of service delivery where the person has to 'fit' the services. This requires listening to people with learning disabilities to find out what they want from services to facilitate choices [41].

## Conclusion

Available data shows that there is a lack of dentists with adequate skills to treat people with disabilities leading to high cost of dental treatment. Thus, we conclude that inconvenient location of dental clinic; lack of dentists willing to treat people with disabilities, attitude of dental staff towards people with learning disabilities were considered to be the barriers and challenges faced for dental health service utilization.
